# An edge-lit volume holographic optical element for an objective turret in a lensless digital holographic microscope

**DOI:** 10.1038/s41598-020-71497-7

**Published:** 2020-09-03

**Authors:** Yeh-Wei Yu, Ching-Cherng Sun, Po-Kai Hsieh, Yi-Hao Huang, Chih-Yuan Song, Tsung-Hsun Yang

**Affiliations:** 1 Department of Optics and Photonics, National Central University, Chung-Li, 320 Taoyüan, Taiwan; 2 Optical Sciences Center, National Central University, Chung-Li, 320 Taoyüan, Taiwan; 3 Department of Electrophysics, National Chiao Tung University, Hsin-Chu, 300 Hsinchu, Taiwan

**Keywords:** Optical sensors, Imaging and sensing

## Abstract

In this paper, we propose and demonstrate the use of an edge-lit volume holographic optical element (EL-VHOE) as a reference waveguide to reduce the volume of a lensless digital holographic microscope. Additionally, a hybrid lensless Fourier transform digital holography is applied to make the EL-VHOE function as an objective turret. It used a spherical wave in the object beam of the EL-VHOE, which served as the reference beam of the microscope. Another sheared spherical wave was used to illuminate the sample. The longitudinal position of the spherical reference beam is changeable. It was shown that the tradeoff between resolution and field of view can be adjusted by changing the longitudinal position of the spherical reference beam. The corresponding experimental results matched the simulational and theoretical predictions. A resolution of approximately 3.11 μm was achieved when the object distance was 6 mm and the longitudinal distance of the spherical reference was 10 mm.

## Introduction

Since the invention of holography^1^, hologram formats have evolved from thin holograms to volume holograms, diffractive/holographic optical element (DOE/HOE) holograms, and digital holograms. Such evolution exploits the resources of modern material science and computer science. However, the essential advantage of holography lies in its wave-front generation capability, and it has proved a useful method to generate a designed or targeted wave-front by diffraction^2–9^. Among these holographic technologies, the volume holographic optical element (VHOE)^10–13^ is special in terms of its optical properties. Strict Bragg conditions lead to holographic multiplexing, i.e., effective diffraction only occurs when the incident wave-front and the wavelength of the reading beam are the same as the reference beam in the recording process^14,15^. Another essential property of volume holography is that it has higher diffraction efficiency than a thin hologram. Theoretically, a 100% diffraction can be achieved if the grating strength is appropriate. These characteristics are extremely useful because VHOE can be applied to optical filtering in spatial or temporal domains and can facilitate modern applications in wave-front transformation^16–18^. Using a VHOE with wave-front transforming and holographic multiplexing provides the potential to build a compact microscope using various lensless imaging techniques. Lensless imaging techniques have demonstrated their superiority in numerous novel applications, such as particle tracking and super-resolution with digital holography^19–26^, digital focusing with optical scanning holography, digital holography, or coding aperture^27–29^, a single-pixel image with orthogonal basis illumination^30–36^, a turbidity image using a computational ghost imaging method^37^, digital holography^38–40^, or speckle correlation^41–43^. A lensless microscope is superior in terms of compact size and offers the potential for multiple applications.

Making a lensless digital holographic microscope (DHM) more compact is a key issue for the commercialization of this technology. Both the lensless Fourier transform digital holographic microscope (LFT-DHM)^19,27,44^ and the lab-on-a-chip imaging are compact in size^45^. However, the trade-off between resolvable resolution and resolvable field of view (FOV) of a DHM is always determined by the pixel size of the image sensor, the object distance, or the laser wavelength. It makes the DHM complicated and not more compact. Therefore, we propose a solution based on a hybrid LFT-DHM (HLFT-DHM). In LFT-DHM, the reference beam of DHM is a divergent spherical wave oriented from the sample plane. In HLFT-DHM, the longitudinal position of the spherical reference beam is changeable. It was shown that the trade-off between resolution and FOV can be adjusted by changing the longitudinal position of the spherical reference beam^46^.

Here, we build an objective turret by combining HLFT-DHM and an edge-lit VHOE (EL-VHOE). The EL-VHOE was used to transform the input beam to the reference beam for the digital hologram, and to select the location of the point source of the reference wave by multiplexing. The introduction of the VHOE made the lensless digital holographic microscope more compact, portable, and useful.

## Design principle

In the design, both the illumination beam of the sample (**Obj**_**ill**_) and the reference beam of the DHM (**R**) were divergent spherical waves. The object was a divergent beam because it enabled the system to spread the diffracted signal (**S**) from the sample to the image sensor. Therefore, the sample could be attached to the top plane of the EL-VHOE to shorten the distance between the sample and the image sensor, thus rendering the system more compact. To match the wave-front of the signal, **R** was also a divergent beam with the virtual point source laterally shearing from the point source of the **Obj**_**ill**_. Two advantages result from this design. The first is to improve the interference fringe period for the image sensor to record the signal at a higher spatial frequency. The other is that the system has become more compact.

**Figure 1:**
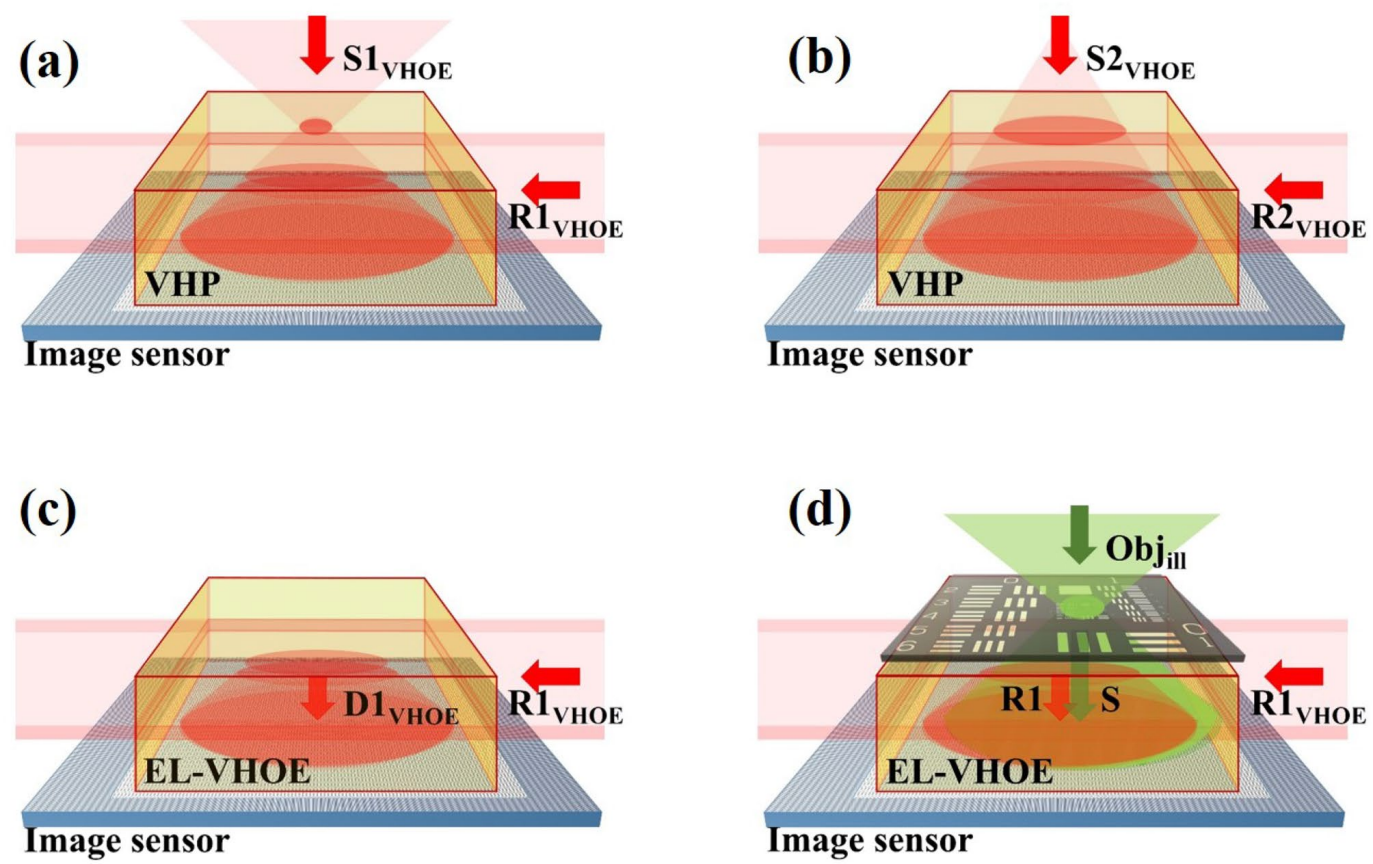
Schematic diagram of a lensless digital holographic microscope with EL-VHOE, and it functions as an objective turret. All beams in the figure have the same wavelength. Here, we use green colour to draw Obj_ill_ to make the picture clearer.

An EL-VHOE is proposed to serve as an objective turret, as illustrated in Fig. 1, where a volume holographic plate (VHP) serves as a recording medium of the EL-VHOE. All wavelengths of the beams in the figure are the same, and we use different colours to make the picture clearer. Figure 1a,b show the recording process of the EL-VHOE; the VHP was used to record a volume hologram. Figure 1a shows the recording of the first hologram. The object beam **S1**_**VHOE**_, a spherical wave with its point source apart from the image sensor in the first distance, was incident on the VHP. The reference beam (**R1**_**VHOE**_) was a light sheet incident on the edge side with the first angle. Figure 1b shows the recording of the second hologram, where **S2**_**VHOE**_ is a spherical wave with its point source apart from the image sensor in the second distance, and **R2**_**VHOE**_) is a light sheet incident on the edge side with the second angle. The VHP then became an EL-VHOE that was used to transform the light sheet (**R1**_**VHOE**_ or **R2**_**VHOE**_) to the corresponding diffraction beam (**D1**_**VHOE**_ or **D2**_**VHOE**_), which had the same wave-front as the object beam (**S1**_**VHOE**_ or **S2**_**VHOE**_). The angular sensitivity of the EL-VHOE is controlled by the thickness of the light sheet and can be expressed as^16^

1 $$I\left( {\vartriangle \theta } \right) = \ell^{2} sinc^{2} \left( {\frac{\ell sin\vartriangle \theta }{\lambda }} \right),$$

where λ is the wavelength of the light source, $\ell$ is the thickness of the EL-VHOE, and $\Delta \theta$ is the angular deviation of the reading beam. Therefore, we can apply angular multiplexing on the EL-VHOE with an acceptable angular tolerance by choosing a proper $\ell$.

Figure 1c shows that we used one of the reference beams (**R1**_**VHOE**_) to read out the corresponding diffraction beam (**D1**_**VHOE**_) with the preferred point source location. The diffraction beam (**D1**_**VHOE**_) served as reference (**R1**) of the DHM, was propagated downward to the image sensor and interfered with the signal (**S**) of the DHM, which was the diffraction beam of the sample placed near the top surface of the EL-VHOE and illuminated by the illumination beam (**Obj**_**ill**_). When the multiplexing of the EL-VHOE is used to choose the distance of the point source of the reference beam, the resolution of the interference fringe between the reference beam and the signal beam can be adjusted. Therefore, the EL-VHOE functions as an objective turret.

The resolvable resolution and resolvable field of view (FOV) of the system depend on how effectively the image sensor can record the interference fringes. By considering: 1. the interference fringe must be larger than twice the resolution of the image sensor; 2. the interference fringe can be detected only when it is located inside the image sensor, the limit of the sample resolution ($\Delta x_{t} )$ and the FOV can be calculated as:

2 $$\Delta x_{t} = {\max}\left( {\left| {1 - \frac{{{\text{zt}}}}{{{\text{zr}}}}} \right|\Delta \xi ,{ }\frac{{\lambda z_{t} }}{N\Delta \xi }} \right),$$

and

3 $${\text{FOV }} = {\text{ max}}\left( {\left| {1 - \frac{{{\text{zt}}}}{{{\text{zr}}}}} \right|{\text{N}}\Delta \xi ,\frac{{\lambda z_{t} }}{\Delta \xi }} \right),$$

respectively, where λ is the wavelength of the light source, z_t_ is the object distance from the sample to the image sensor, N is the one-dimensional pixel number of the image sensor, and Δξ is the pixel size of the image sensor. Therefore, we can change the FOV and resolution by changing z_r_. If z_r_ is the same as z_t_, HLFT-DHM becomes LFT-DHM. We obtain the smallest resolution (Δx_*f*_) and the smallest FOV:

4 $$\Delta x_{f} = \frac{{\lambda z_{t} }}{N\Delta \xi },$$

and

5 $$FOV = \frac{{\lambda z_{t} }}{\Delta \xi },$$

respectively. For conventional LFT-DHM, z_r_ is not changeable, and the resolution and field of view (FOV) are both controlled by the object distance z_t_. Therefore, because of the object distance and the moving mechanism the lensless microscope was no longer compact.

If the reference beam of DHM (**R**) is a collimated plane wave, which means z_r_ approaches infinity, this causes a small interference fringe period when the fine structure of the sample is recorded. The limited pixel size of the image sensor made it impossible to record these types of fringes. As a result, we obtain a large resolution $\Delta \xi$ and a large FOV N $\Delta \xi$.

## Simulation demonstration

**Figure 2:**
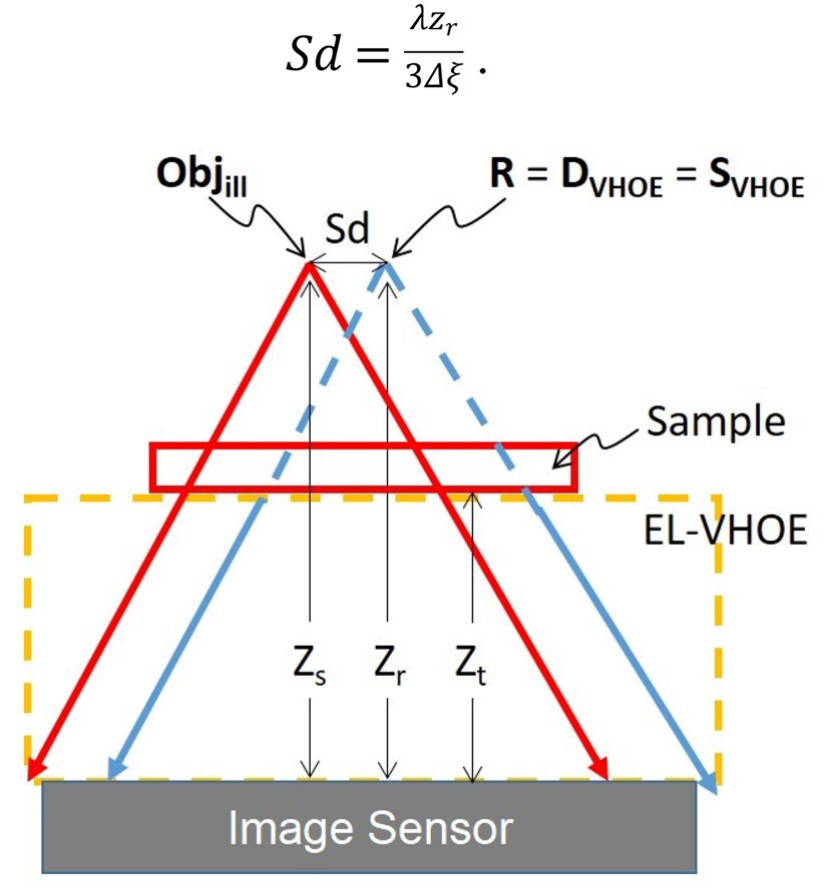
Geometry of the new design when point sources are introduced.

Here, we build a simulation model for the EL-VHOE-based HLFT-DHM without considering the issue of Bragg mismatch. Therefore, we can focus on demonstrating the trade-off between the resolution and FOV and determining the performance limit of the proposed objective turret. As illustrated in Fig. 2, ***z***_***s***_ and ***z***_***r***_ are the distances from the image sensor to the point source of **Obj**_**ill**_ and **R**, respectively, and ***z***_***t***_ is the distance from the image sensor to the sample. The shearing distance (*Sd*) was set to remove aliasing noise. Therefore, in the Fourier domain, the 1st order signal is located between the DC term noise and the duplication of the twin image noise. And it was expressed as:

6 $$Sd = \frac{{\lambda z_{r} }}{3\Delta \xi }.$$

The simulation area in each plane is 10 mm × 10 mm, and the simulation resolution is 0.5 µm. We apply the two Fast Fourier Transform methods to simulate the beam propagation by the angular spectrum beam propagation method^47^; this is written as:

7 $${\text{ASBP}}\left\{ {E_{0} \left( {{\text{x}},{\text{y}}} \right);{\text{z}}} \right\} = F^{ - 1} \left\{ {F\left\{ {E_{0} } \right\}\left[ {k_{x} ,k_{y} } \right]exp\left[ {i\sqrt {k^{2} - k_{x}^{2} - k_{y}^{2} } z} \right]circ\left( {\frac{{\sqrt {k_{x}^{2} + k_{y}^{2} } }}{k}} \right)} \right\}\left[ {x,y} \right],$$

where E_0_ is the input electrical field, λ is the wavelength, d is the propagation distance, k is the wavenumber, and k_x_ and k_y_ are the components of the wave vector along the x-axis and y-axis, respectively. The circle function is applied to filter out the evanescent wave. In order to provide sufficient sampling in the spectrum domain, the matrix should be zero-padded to a matrix of double size. The maximum matrix size after zero padding is up to 40,000 × 40,000 in size. A computer with 196 GB RAM was used to support the maximum matrix size in the simulation. Signal **S** was simulated as a diffracted beam of the sample illuminated by **Obj**_**ill**_ and propagated to the image sensor plane.

8 $$S\left( {{\text{x}},{\text{y}}} \right) = {\text{ASBP}}\left\{ {Sample\left( {{\text{x}},{\text{y}}} \right)Obj_{ill} \left( {{\text{x}},{\text{y}}} \right);{\text{z}}_{t} } \right\},$$

Reference R directly illuminated the image sensor.

9 $$R\left( {{\text{x}},{\text{y}}} \right) = {\exp}\left[ {ikr_{R} \left( {{\text{x}},{\text{y}}} \right)} \right],$$

**Figure 3:**
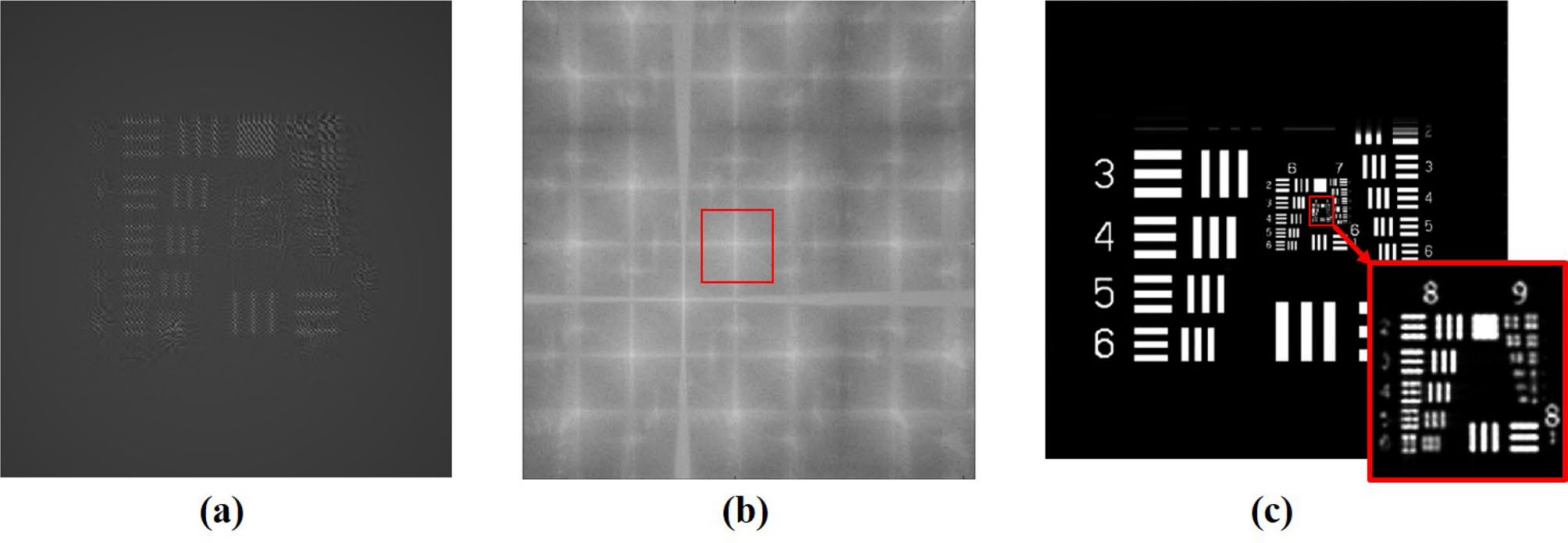
Simulated images: (**a**) on the image sensor plane, (**b**) on the point source plane by backward propagation based on (**a**), and (**c**) on the plane of the USAF target.

In Fig. 3, the distances ***z***_***s***_ and ***z***_***r***_ were both set as 8 mm, ***z***_***t***_ was limited by the thickness of the VHOE and was set as 6 mm, the pixel size of the image sensor was 3.75 μm × 3.75 μm, the number of pixels was 1,280 × 960, and the lateral dimensions were 4.8 mm × 3.6 mm. The interference fringes of **S** and **R** on the image sensor are illustrated in Fig. 3a.

10 $$I\left( {{\text{x}},{\text{y}}} \right) = \left| {R\left( {{\text{x}},{\text{y}}} \right) + S\left( {{\text{x}},{\text{y}}} \right)} \right|^{2} .$$

We integrated the illuminance of each pixel to obtain the signal detected by the image sensor. Then, we used the interpolation method to reconstruct the image of the HLFT-DHM. Each pixel of the detected signal was replaced by a 7 × 7 matrix with the same value to obtain the expanded matrix of the detected signal I_exp_(x, y), which sets the pixel resolution of the reconstruction image to 0.54 µm. The expanded matrix I_exp_(x, y) multiplied the field of **R** $\left( {{\text{x}},{\text{y}}} \right)$ on the image sensor and then backpropagated to the plane of the point source of both **R** and **Obj**_**ill**_,

11 $$E_{spectrum} \left( {{\text{x}},{\text{y}}} \right) = {\text{ASBP}}\left\{ {R\left( {{\text{x}},{\text{y}}} \right)I_{exp} \left( {{\text{x}},{\text{y}}} \right); - {\text{z}}_{r} } \right\}.$$

The result is illustrated in Fig. 3b, where the first-order diffraction pattern can be separated. Therefore, the twin image noise is mitigated. After the system separated the first-order diffraction signals (see the rectangular window with a red border in Fig. 3b), it propagated forward to the sample plane to reconstruct the image,

12 $$Image\left( {{\text{x}},{\text{y}}} \right) = {\text{ASBP}}\left\{ {E_{spectrum} \left( {{\text{x}},{\text{y}}} \right)Filter\left( {{\text{x}},{\text{y}}} \right);{\text{z}}_{r} - {\text{z}}_{t} } \right\}.$$

The recovery image on the sample plane is illustrated in Fig. 3c. We found that the smallest resolvable element was element 5 in group 8 (G8/E5). The corresponding resolution was 1.23 µm.

**Figure 4:**
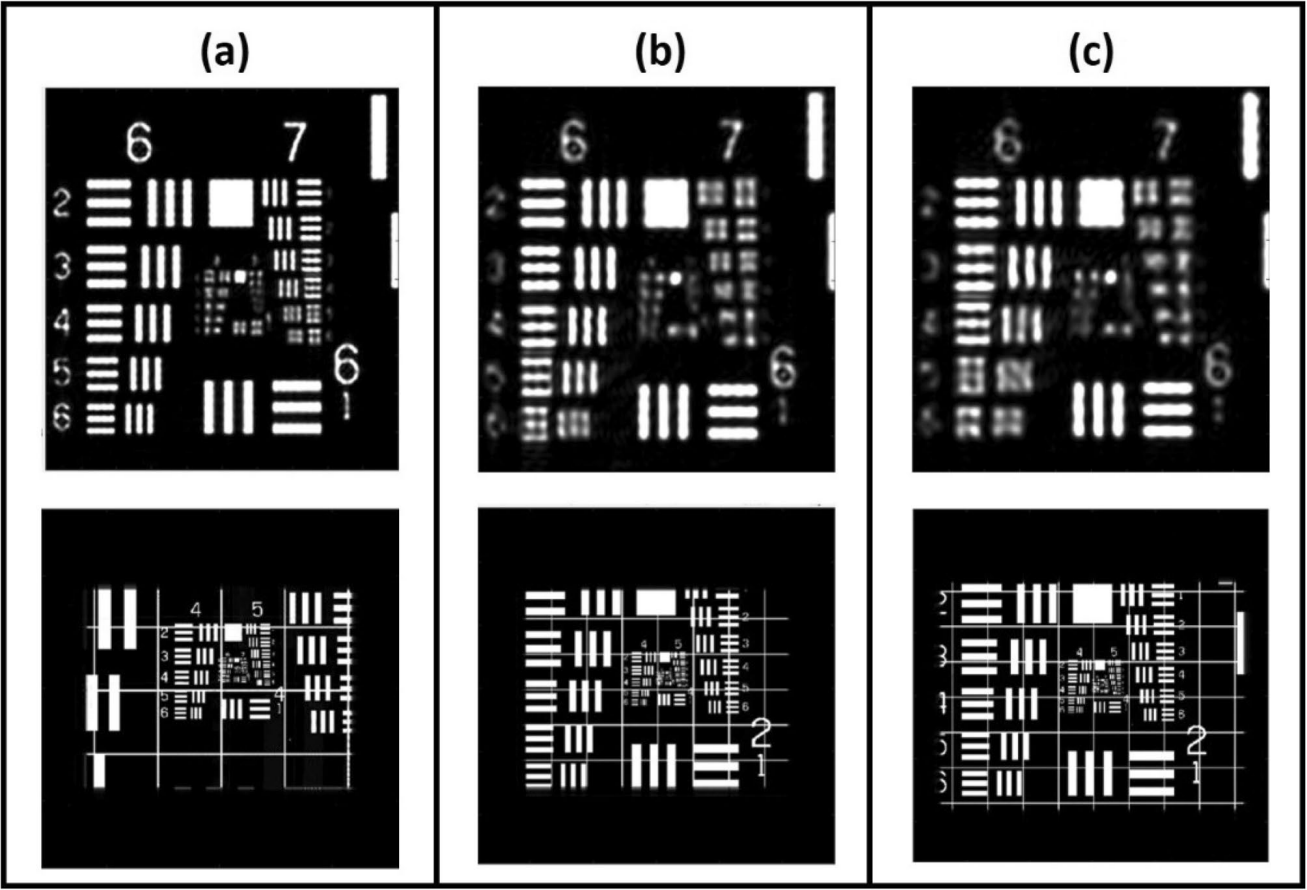
Simulation result for the resolution (top row) and FOV (bottom row), where the reference beam distance is equal to (**a**) 10 mm, (**b**) 20 mm, and (**c**) 40 mm.

However, limited by the experimental setup, the shortest ***z***_***r,***_ and ***z***_***s***_ are 10 mm. To simulate the performance limit of the proposed objective turret, we fixed ***z***_***t***_ at 6 mm and equally changed ***z***_***r,***_ and ***z***_***s***_ as 10 mm, 20 mm, and 40 mm. The simulation results are illustrated in Fig. 4. When ***z***_***s***_ was small, the divergent angle of the spherical wave on the image sensor was large. This means that the signal **S** could then be recorded at a high spatial frequency, which is a clear advantage. However, there is a limit to both the pixel size and the sensor of the image sensor. This led to the limitation of the FOV. Figure 4a shows that when z_s_ is 10 mm, the smallest resolvable element is G7/E4 (top row), and the FOV is 1,550 µm × 2,102 µm (bottom row). Figure 4b shows that when z_s_ is 20 mm, the smallest resolvable element is G6/E6 (top row), and the FOV is 3,680 µm × 2,756 µm (bottom row). Figure 4c shows that when z_s_ is 30 mm, the smallest resolvable element is G6/E4 (top row), and the FOV is 4,276 µm × 3,206 µm (bottom row).

## Experimental demonstration

**Figure 5:**
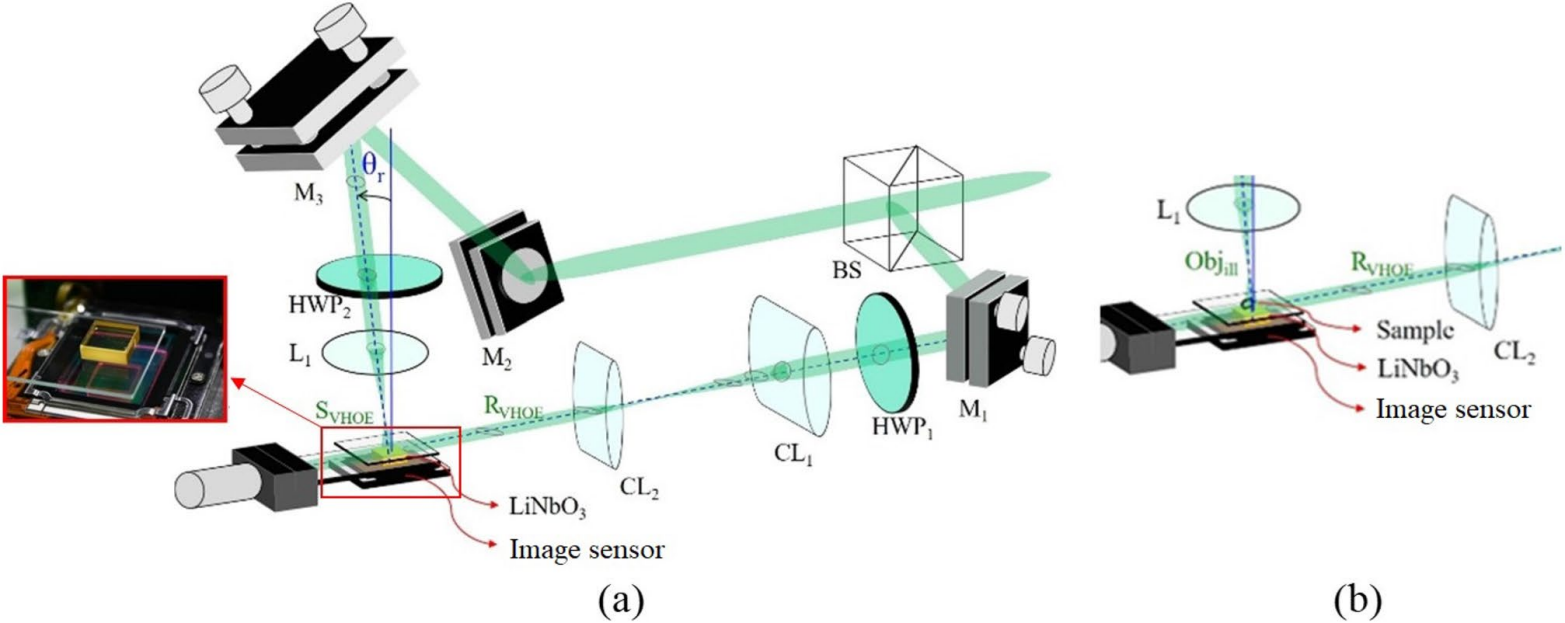
Optical setup for (**a**) constructing the VHOE, and (**b**) operating the DHM.

In the experiment, we used a photorefractive crystal (Fe:LiNbO_3_, with dimensions of 1 cm × 1 cm × 0.5 cm, as a VHP of the EL-VHOE (Fig. 5). A 532-nm continuous-wave laser (Verdi-V5, Coherent Inc., coherent length 50 m) was used as the light source. The coherent length of the light source had to be long enough to ensure good visibility of the interference fringe in both the EL-VHOE recoding operation and the DHM operation. The sample, a USAF target, was attached to the top surface of the EL-VHOE. The distance between the sample and the image sensor (z_t_) was 6 mm. The laser beam was separated into two beams.

Figure 5a shows the optical setup of the EL-VHOE construction process. The first beam, which served as the **S**_**VHOE**_, was incident normally (θ_r_ = 0), passed through the 100 × objective lens (L_1_), and produced a focal point for the signal of the EL-VHOE (**S**_**VHOE**_). The second beam, which served as the **R**_**VHOE**_, was shaped like a light sheet by the cylindrical lenses set CL_1_ and CL_2_, and then illuminated the VHP horizontally. The interference fringe of **R**_**VHOE**_ and **S**_**VHOE**_ was recorded by the VHP to construct the EL-VHOE. The optical power of the **R**_**VHOE**_ and **S**_**VHOE**_ are both 120 mW. The recording time was 1 min.

**Figure 6:**
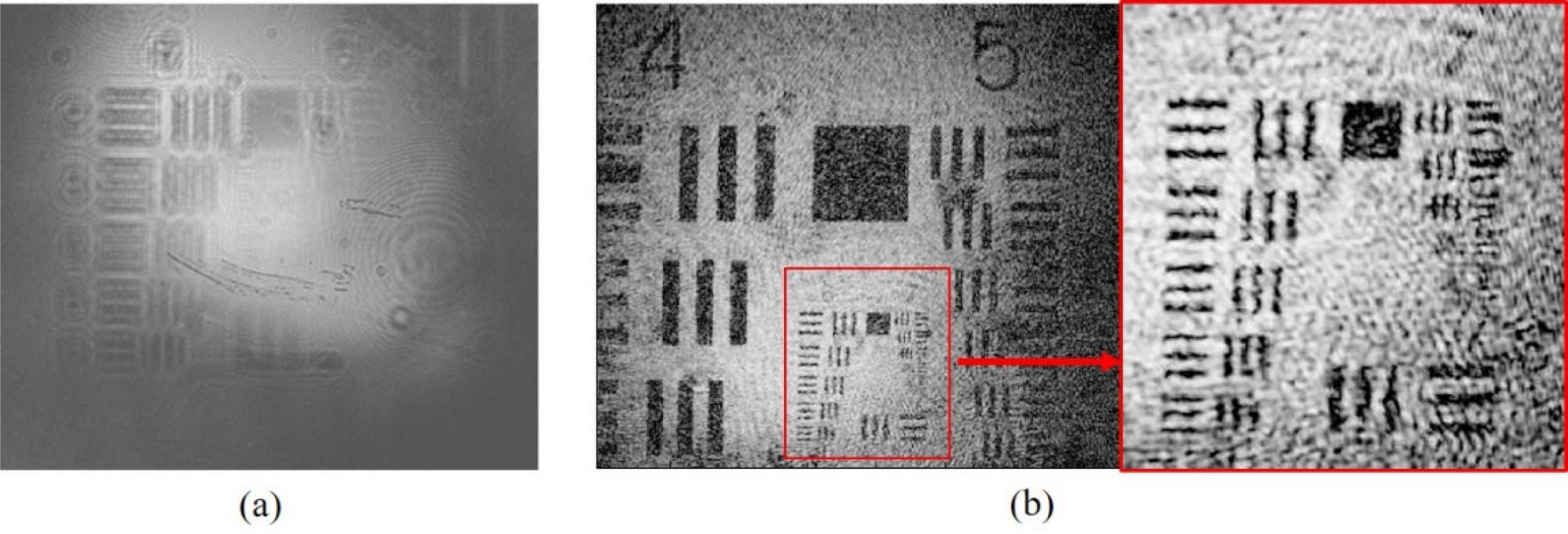
Experimental results for (**a**) the interference fringes caught by the image sensor, and (**b**) retrieved image of the USAF target, where G7/E3 can be resolved.

**Figure 7:**
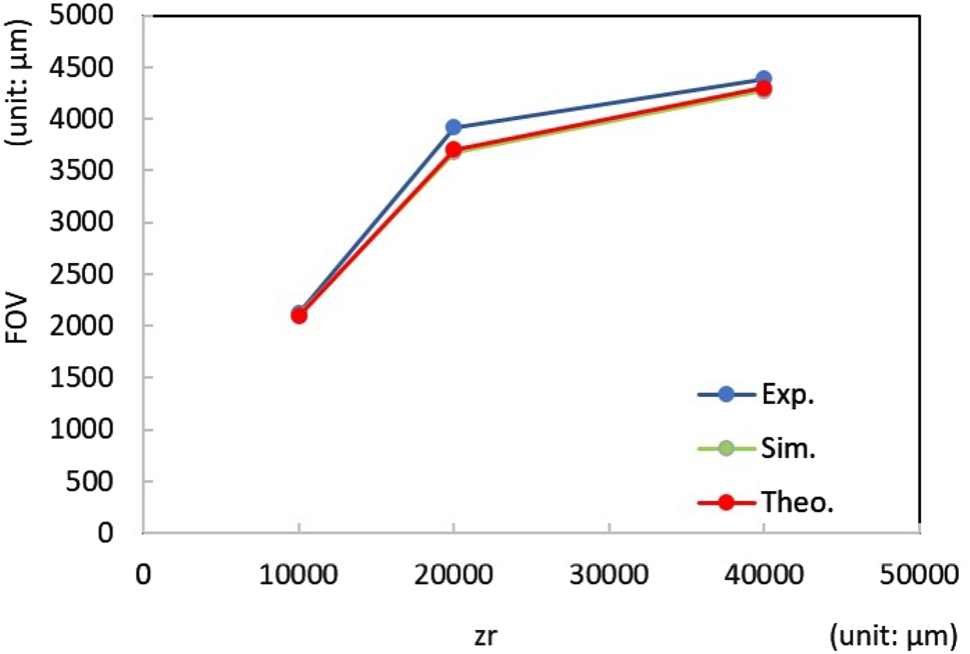
Comparison chart of FOV on the long side with variant zr. The blue line is the experimental result, the green line is the simulation result, and the red line is the theoretical prediction using Eq. (3).

Figure 5b shows the optical setup for the operation of the DHM. L_1_ was shifted a distance (Sd), according to Eq. (3) to produce an **object**. The **Obj**_**ill**_ illuminated the sample to produce the diffraction signal **S**. The **R**_**VHOE**_ illuminated the EL-VHOE to produce the **D**_**VHOE**_. **D**_**VHOE**_ served as reference **R** of the DHM, and it interfered with the signal **S** on the image sensor plane. The optical power of **R**_**VHOE**_ and **Obj**_**ill**_ are 25 mW and 15 µW, respectively. Figure 6 shows the experimental results for both z_r_ and z_s_ chosen as 10 mm. The interference fringes of **R** and **S** are captured by the image sensor (daA1280-54um, Basler, pixel size 3.75 µm, pixel number 1280 × 960), as shown in Fig. 6a. The fringe was digitally inverse propagated to the plane of the focus point, and the first-order signal was separated. Then, it was propagated digitally to the plane of the sample to reconstruct the image. By digital noise filtering, we obtained the reconstructed image, as shown in Fig. 6b, where the vertical fringe of group 7/element 3 (G7/E3) can be resolved. The corresponding resolution is 3.11 µm and is smaller than the pixel size of the image sensor. Some scattering noise and interface reflection noise caused by the EL-VHOE limited the image quality to marginally less than that of the simulation result. The experimental results of the FOV for z_r_ equal to 10,000, 20,000, and 40,000 is 1,870 µm × 2,117 µm, 2,993 µm × 3,920 µm, and 3,320 µm × 4,388 µm, respectively. Figure 7 shows the comparison chart of the FOV on the long side with variant z_r_, where ***z***_***s***_ is always the same as ***z***_***r***_. The blue line represents the experimental result, the green line represents the simulation result, and the red line shows the theoretical prediction using Eq. (3). It confirms that the FOV in the experiment is approximately the same as that in the simulation result and the theoretical prediction.

## Conclusion

In this paper, we propose and demonstrate the use of an edge-lit volume holographic optical element (VHOE) as a reference waveguide to reduce the volume of an LFT-DHM, and as an objective turret to change the image magnification. The object beam of the EL-VHOE **(S**_**VHOE**_) was reconstructed by illumination from the edge (**R**_**VHOE**_) and then became the reference beam (**R**) during the operation of the microscope. An HLFT-DHM is applied to enable the function of an objective turret. It used a spherical wave in the object beam of the EL-VHOE, which served as the reference beam of the microscope. Another sheared spherical wave was used to illuminate the sample. The benefit of this design is that the spherical wave provides a slanted ray that fits the light angle of the signal with a higher spatial frequency. Therefore, for such information, the period of the interference fringe is long enough to be recorded by the image sensor. Besides, the longitudinal position of the spherical reference beam (**R**) is changeable. It was shown that the trade-off between resolution and FOV can be adjusted by changing the longitudinal position of the spherical reference beam (**R**).

For theoretical analysis, we derive equations to analyse the resolution limit and the FOV limit of the objective turret by considering: 1. that the interference fringe must be larger than twice the resolution of the image sensor; 2. that the interference fringe can be detected only when it is located inside the image sensor. We also built a simulation model based on the angular spectrum beam propagation method. The simulation results show that we can trade resolution with FOV by changing the longitudinal depth of the reference beam (**R**).

Finally, we constructed an experimental setup to demonstrate the feasibility of the proposed method. For the FOV, the experimental results matched the simulation predictions and theoretical predictions. A resolution of approximately 3.11 μm was achieved when the object distance was 6 mm and the longitudinal distance of the spherical reference was 10 mm. In summary, the EL-VHOE reduced the system volume, and its function as an objective turret was demonstrated.
